# Scaling Up Magnetic Nanobead Synthesis with Improved Stability for Biomedical Applications

**DOI:** 10.1021/acs.jpca.2c05902

**Published:** 2022-12-16

**Authors:** Nadja C. Bigall, Marina Rodio, Sahitya Avugadda, Manuel Pernia Leal, Riccardo Di Corato, John S. Conteh, Romuald Intartaglia, Teresa Pellegrino

**Affiliations:** † Istituto Italiano di Tecnologia, Via Morego 30, 16163 Genova, Italy; ‡ Leibniz Universität Hannover, Callinstrasse 3A, 30167 Hannover, Germany; § Universidad de Sevilla, Facultad de Farmacia, Departamento de Química Orgánica y Farmacéutica, c/Profesor García González, 2, 41012 Sevilla, Spain; ∥ CNR, Institute for Microelectronics and Microsystems (IMM), Via Monteroni, Lecce 73100, Italy

## Abstract

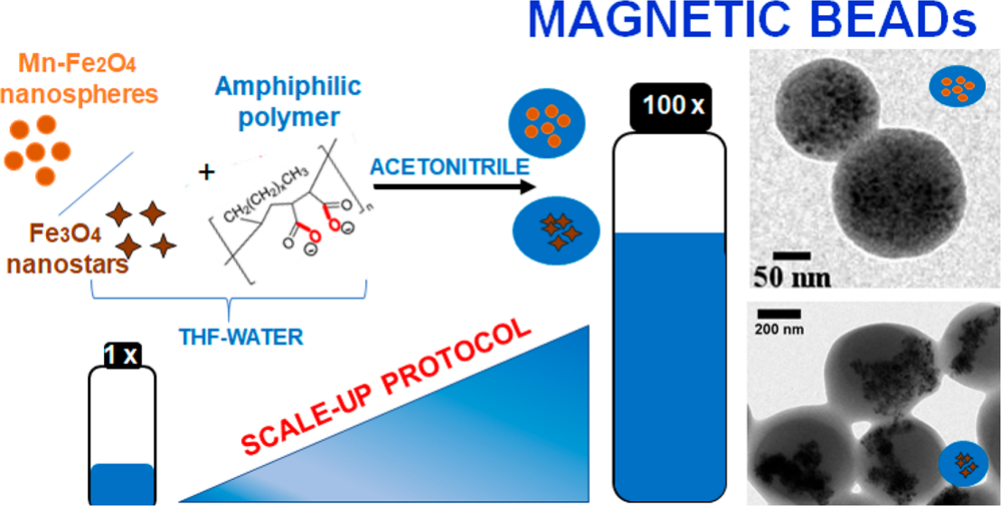

The growing interest in multifunctional nano-objects based on polymers and magnetic nanoparticles for biomedical applications motivated us to develop a scale-up protocol to increase the yield of polymeric magnetic nanobeads while aiming at keeping the structural features at optimal conditions. The protocol was applied to two different types of magnetic ferrite nanoparticles: the Mn-ferrite selected for their properties as contrast agents in magnetic resonance imaging and iron oxide nanostar shaped nanoparticles chosen for their heat performance in magnetic hyperthermia. At the same time, some experiments on surface functionalization of nanobeads with amino modified polyethyelene glycol (PEG) molecules have provided further insight into the formation mechanism of magnetic nanobeads and the need to cross-link the polymer shell to improve the stability of the beads, making them more suitable for further manipulation and use. The present work summarizes the most important parameters required to be controlled for the upscaling of nanobead synthesis in a bench protocol and proposes an alternative cross-linking strategy based on prefunctionalization of the polymer prior to the nanobead formation as a key parameter to improve the nanobead structural stability in solutions at different pHs and during surface functionalization.

## 1 Introduction

Over the past decade, the progress in synthesis of magnetic nanostructures has been accompanied by a parallel exploitation of these systems in various research fields; among them, biology and medicine have gained significant benefits.^1^ In nanomedicine, iron oxide nanoparticles (IONPs) are the most applied metal oxide based materials for imaging and therapeutic applications thanks to their properties of superparamagnetism, chemical stability, biocompatibility and *in vivo* biodegradability.^2^ For example, colloidal superparamagnetic nanoparticles are already FDA approved and commercially available as contrast agents in magnetic resonance imaging, especially for the T_2_ and T_2_* transversal relaxivity detection modes.^3^ Another example involves their use in delivery of genetic materials to living cells in the presence of a magnetic field gradient (“magnetofection”).^4^ In the meantime, several groups have reported how the controlled aggregation of several superparamagnetic nanoparticles within a single matrix of different nature, such as polymer, silica, or fat droplets, results in the so-called “magnetic nanobeads”, which have certain advantages compared to single coated individual nanoparticles.^5^ These magnetic nanobeads made of multiple magnetic nanoparticles have indeed faster magnetophoretic mobility with respect to single nanoparticles.^6^ This effect arises from the cumulative magnetic moments of all individual particles while the nanoobject remains mostly superparamagnetic. Several groups have proposed various nanobead preparation and functionalization methods to make multitasking materials exploitable in preclinical studies for diagnosis and treatment of diseases.^7,8^ Also in our group we have developed a robust method for the synthesis of nanobeads with control over core and surface properties, and we have tested them as nanoplatforms in several *in vitro* applications.^9−11^ To mention some examples of applications, by decorating our magnetic nanobeads with a thermoresponsive polymer, we were able to load and release chemo-therapeutic drugs entrapped in the polymer.^12^ Here the release was based on a change in the temperature of the solution from 37 °C to 47 °C.^12^ On the other hand, by tuning the electrostatic interactions between the polymeric shell and oligonucleotides (siRNA), we could deliver and release siRNAs material into living cells and perform protein silencing experiments (in the specific case by using anti-GFP RNA).^13^ By our previously developed methods, only a few micrograms of magnetic material could be prepared, and we were able to provide proof of concept on *in vitro* study with living cells.^14,15^ For animal experiments these doses are significantly low and scale-up approaches would be required for a feasible *in vivo* studies. Also, these applications require beads of very high quality with respect to certain specific properties. A system aimed as a probe for *in vitro* applications must have an appropriate size for selectively binding to a single cell and a high magnetization and magnetophoretic mobility that allows a quick response (within few minutes) of the magnetized cells to a permanent magnet of lab use (0.5 T).^9^ Furthermore, on the one hand, an appropriate polymer thickness is required to preserve the bead structure, preventing leakage of the nanoparticles, while allowing at the same time facile molecular functionalization of the bead surface. In addition, the polymer layer should not be too thick to cushion the response to magnetic fields. All these parameters are easily controllable by our previously developed nanobead fabrication processes in the small scale. Moreover, these protocols are straightforward since it only depends on a coprecipitation of a commercially available amphiphilic polymer, namely, poly(maleic-*alt*-1,1-octadecene) (PMA-OD) together with the nanoparticles prepared by common thermal decomposition synthesis, which do not even need to be surface modified prior to inclusion in the polymer. The choice of the PMA-OD polymer to assemble the magnetic nanoparticles into the beads is also dictated by its amphiphilic nature. Indeed, the interdigitation of the alkyl chains of the octadecene with the nonpolar surfactants at the nanoparticles surface (especially when using nanoparticles prepared by nonhydrolytic methods) favors the polymer interaction with the magnetic nanoparticles during the assembly, while the hydrophilic polymer backbone ensures the water stability of the beads, once formed, by charge repulsions.^10,16^ At the same time, this polymer being commercially available enables widespread use of our scale-up protocol since no specific polymer chemistry skills are needed to handle this on-shelf available polymer. Moreover, to improve the magnetic response, even the choice of the magnetic nanoparticles to include into the beads becomes relevant. With our methods we could prove in different works the encapsulation of different types of magnetic nanocrystals and thus the different magnetic responses.^9,15,17−19^

However, for our preparation route, two main challenges remain to be addressed. The first one is the scalability of the protocol. The second concerns the colloidal stability and the structural integrity of our nanobeads in saline solution or upon further surface functionalization of the nanobeads. The availability of a scale-up approach would facilitate the use of these magnetic materials *in vivo* in animal experiments and in all those biomedical applications by which the *in vitro* proof of principle study (*i.e.*, gene delivery) was provided. So far, our nanobeads preparation method employed 70 μg in iron of magnetic nanoparticles for a solution volume of 1 mL. While under these optimized conditions we were able to synthesize acceptable quality nanobeads, for obtaining larger batches while maintaining the same quality of the nanobead batch, we repeat manually the same synthesis protocol in a 4 mL vial several times and then magnetically collect the beads and subsequently merge the different batches together in one single vial. This small-scale protocol is laborious and time-consuming. In the present article, we report a one-pot upscaling approach that enables us to obtain 7 mg (nominal amount) of iron as magnetic beads and hence to achieve an approximately 100-fold increase of the amount of magnetic nanobeads per batch. We achieve the scale-up of high-quality magnetic beads by improving the control over certain synthesis parameters. Indeed, the coprecipitation method employed to prepare nanobeads was here found to be sensitive to the humidity of the air as well as to the water content of the solvent. All these parameters affect the hydrolyses of the maleic anhydride groups and hence the solubility of the polymer in the reaction media with an effect on the nanobead size and morphology. In the present work, by controlling the amount of water present in the synthesis done under air-free conditions, we managed to circumvent these problems and hence enabled scaling up the nanobead synthesis in one single reaction batch.

The second drawback of our previous synthesis method was the solution stability related to the charge repulsions of the carboxyl groups present on the nanobead surface: upon exposure to saline solution, the high ionic strength shields the negative charges on the nanobeads surface with consequent precipitation. To further stabilize the nanoparticles in a charge-free manner, poly(ethylene glycol) (PEG) polymer molecules could be attached to the nanobead surface, which provide steric repulsion between nanobeads and enhance their colloidal stability.^9^ After modification of our nanobeads with PEG, they were stable in normal buffer solutions as it was confirmed by, *e.g*., dynamic light scattering. However, presumably due to the chemical coupling procedure which is performed *via* amide bond formation in the presence of 1-ethyl-3-(3-(dimethylamino)propyl)carbodiimide (EDC), we observe a partial swelling or unfolding of the polymer from the nanobeads in a way that the polymer shell density in our TEM images is either thinned significantly or not “visible” anymore. Since in a variety of experiments a defined polymer shell is requested, *e.g*., for further functionalization and for shielding the nanoparticles from their environment, the observed effect can be to some extent problematic. In the present article, we report about a modified PEG prefunctionalization step that ensures a better nanobead solution stability, making them available for next *in vivo* applications.^20^ In summary, this work suggests solutions to two major problems currently found in the synthesis of superparamagnetic nanoparticle based nanobeads produced with amphiphilic polymers like our commercially available poly(maleic anhydride-*alt*-1-octadecene): first, we suggest a method leading to 2 orders of magnitude higher amounts of nanobeads than previously reported. Next, we report on how to lead to colloidal and structurally stable polymeric nanobeads even at pH values close to the polymer isoelectric point, when using amino PEG molecules and additional diamines derivates. Furthermore, the scale-up protocol of magnetic nanobeads was implemented with two types of ferrite magnetic nanoparticles: the manganese iron oxide nanoparticles of composition MnFe_2_O_4_ and size 8.5 nm usable as contrast agents in magnetic resonance imaging (MRI)^21^ and the iron oxide nanostars of composition Fe_2_O_3_ and size 13 nm exploitable as heat mediators in magnetic hyperthermia.

## 2 Materials and Methods

### Chemicals

Poly(maleic anhydride-*alt*-1-octadecene), Mn 30,000–50,000 (Aldrich), Milli-Q water (18.2 MΩ, filtered with filter pore size 0.22 μM) from Millipore, acetonitrile (HPLC grade, J. T. Baker) and tetrahydrofuran anhydride (Carlo Erba, p.a.), iron oxide hydroxide (Sigma-Aldrich, #371254), iron acetylacetonate (Sigma-Aldrich, 99%), manganese acetylacetonate (Sigma-Aldrich, #245763), hexadecanediol (Sigma-Aldrich, 90%), dodecylamine (Sigma-Aldrich, 98%), lauric acid (Sigma-Aldrich, 99%), benzyl ether (Sigma-Aldrich, 98%), and octadecene (Sigma-Aldrich, 90%), as well as 1-ethyl-3-(3-(dimethylamino)propyl)carbodiimide hydrochloride (EDC) (Aldrich, commercial grade), *O*-(2-aminoethyl)-*O*′-methyl polyethylene glycol (“aminoPEG750”, Aldrich), *N*,*N*-dimethylethylenediamine (for “tertiary amine functionalization”, Aldrich), 2-(2-pyridyl)ethylamine (for “pyridine functionalization”, Aldrich), and 2,2′-(ethylendedioxy)bis(ethylamine) (referred to in this article as “diamine” Aldrich), tetramethylammonium hydroxide (TMAOH, Sigma-Aldrich) were used without further purification. Acetonitrile (ACN, not anhydrous) was purchased from J. T. Baker and stored in the glovebox.

### Synthesis of Manganese Iron Oxide Nanoparticles (Mn-IONPs)

To perform the procedure of synthesis of the beads, a sample composed of MnFe_2_O_4_ of 8.5 ± 1.5 nm was used. The sample was synthesized according to the procedure reported in previous works.^10,13,22^ Briefly, 1 mmol of manganese acetylacetonate, 2 mmol of iron acetylacetonate, 10 mmol of hexadecanediol, 6 mmol of dodecylamine, 6 mmol of lauric acid, and 20 mL of benzyl ether was mixed and heated to 140 °C for 1 h while stirring under a flow of nitrogen. Then, the solution was heated to 210 °C for 2 h and subsequently to 300 °C for 1 h. The reaction was washed several times using ethanol, acetone, and isopropanol in combination with centrifugation and subsequent redispersion in toluene. The resulting Mn-IONPs were dissolved in toluene solvent and stored at room temperature.

### Standard Synthesis of Nanobeads

For the standard synthesis, the polymer solution of poly(maleic-*alt*-1octadecene) (PMA-OD) in THF (50 mM) was prepared under air following an adapted method from previously reported routes.^17^ Briefly, in an 8 mL vial, the Mn-IONPs in toluene (2.2 μL of a Mn-IONP solution at 31,8 g_(Fe+Mn)_/L measured by ICP) were dried under a nitrogen flow and 12 μL of the polymer PMA-OD (50 mM) was added with addition of THF to a final volume of 200 μL. The open vial was shaken at room temperature for 1 h at 1000 rpm. Subsequently, 800 μL of ACN was added with a constant flow rate of 0.25 mL/min within approximately 3 min. Finally, the nanobeads were collected to the wall of the vial upon exposure to a 0.3 T commercial neodymium magnet. After discarding the THF/ACN solvent, the magnetic nanobeads sample was dissolved in 1 mL of deionized water.

### Modified Synthesis Route of Nanobeads under Anhydrous Conditions

In order to prevent aging of the polymer, the polymer powder was stored under inert gas conditions in a glovebox. In the glovebox, a fresh stock solution of polymer was prepared by dissolving 175 mg of PMA-OD in 10 mL of anhydrous THF (50 mM in monomer unit concentration). 2.2 μL of a Mn-IONP solution at 31.8 g_(Fe+Mn)_/L, as measured by ICP, in toluene (8.5 nm in diameter) was added to a vial and dried on a magnet (without any septum) under a slow flow of nitrogen. Then, the vial was transferred into the glovebox under a nitrogen atmosphere. 12 μL of the THF anhydrous PMA-OD solution (50 mM in monomer unit) was added to the vial. The vial was closed and secured by means of a septum and parafilm and taken from the glovebox. Then, 0 μL, 2.5 μL (139 μmol), 5 μL (278 μmol), or 7.5 μL (417 μmol) of deionized water was added to each vial of polymer and Mn-IONPs in THF at a rate of 0.8 mL/min with a syringe. Next, the THF volume was adjusted to each vial in order to have constant volumes of Mn-IONPs+PMA-OD solution+THF+water = 200 μL. In more detail, 188 μL, 185 μL, 183 μL, or 180.5 μL of THF was added to each vial so that the reaction volume, in each vial, reached 200 μL. The closed vial was vortexed for 1 h at 1000 rpm, followed by the addition of 800 μL of ACN at a flow rate of 250 μL/min (maintaining a vortex rate of 1000 rpm during ACN addition).

### Modified Synthesis Route for Scale-Up Production of Nanobeads

#### 20× Scale-Up

For upscaling the synthetic approach 20 times, in a 20 mL vial, 44 μL of Mn-IONP solution in toluene at 31.8 g_(Fe+Mn)_/L as measured by ICP was placed in an open vial and dried under a mild low nitrogen flux. Subsequently, the open vial was dried in a glovebox under a nitrogen atmosphere (MBraun, H_2_O < 0.1 ppm; O_2_ < 0.1–0.5 ppm). Under similar dry and oxygen-free inert gas conditions, 240 μL of PMA-OD (50 mM) dissolved in anhydrous THF was added and a clear brown solution was formed. The vial was closed and secured by means of a septum protected by parafilm. The closed vial was transferred out of the glovebox, and by means of a syringe pump, a defined water volume was added with a flow rate of 0.8 mL/min. Four different volumes (0 μL, 50 μL, 100 μL, and 150 μL) of deionized water were added to each vial containing the PMA-OD polymer and Mn-IONPs in THF so that the water molecules/polymer monomer unit ratio was set at 0, 232, 463, or 695, respectively. For the 20-fold scale-up, the THF volume to add to each vial was adjusted to the water volumes, in order to yield a total volume of Mn-IONPs+PMA-OD+THF+water = 4 mL in all cases. Hence, for dissolving the nanoparticles and the polymer, THF volumes of 3.760 mL, 3.710 mL, 3.660 mL, and 3.610 mL were added to the vial containing 50 μL (sample 20×_1), 100 μL (sample 20×_2), and 150 μL (sample 20×_3) water, respectively. The closed vial was vortexed for 45 min at 1000 rpm, followed by the addition of 16 mL of ACN at a flow rate of 5 mL/min (maintaining a vortex rate of 1000 rpm).

#### 40×, 80×, and 100× Scale-Up

40×, 80×, and 100× upscaling protocols were made by following exactly the same scaling up protocol as in the 20-fold procedure where the water, Mn-IONP, and polymer amounts were properly scaled by a factor of 40, 80, or 100 rather than 20 times, while the reaction volume of Mn-IONPs+PMA-OD+water+THF was kept below 6 mL and the ACN volume was kept at 16 mL in order to always yield a final volume of 20 mL.

For a practical example, in a typical 100-fold scale-up protocol, 220 μL of Mn-IONP solution in toluene at 31.8 g_(Fe+Mn)_/L as measured by ICP was dried under a nitrogen flow and 1200 μL of anhydrous 50 mM PMA-OD was mixed with anhydrous THF. Deionized water was added at a well-defined volume of 0 μL (no water added), 250 μL, 500 μL, or 750 μL at a rate of 8 mL/min, followed by addition of THF and 45 min of vortexing at 1000 rpm. The THF amounts added for each reaction were respectively 3.760 mL, 3.710 mL, 3.660 mL, or 3.610 mL. Next, the final addition of 16 mL of ACN at a rate of 5 mL/min (in order to keep constant the injection time of 200 s in total) was performed.

#### Scale-Up Production of Nanobeads When Using Iron Oxide Nanostars

For the production of nanobeads made with iron oxide nanostars (IONS of 13 ± 1 nm size), 313 μL of a CHCl_3_ solution of IONS (8.4 mg_Fe_/mL corresponding to 2.6 mg of Fe) was added to a 40 mL glass vial containing 7 mg of decanoic acid (DA, 100 ligands/nm^2^). This solution was sonicated at 60 °C for 10 min in a sealed vial kept under nitrogen. After drying the sample under a gentle nitrogen flux, 1.2 mL of PMA-OD (50 mM in anhydrous THF) was added and the mixture was sonicated for 1 min at 60 °C. Next, 3.66 mL of anhydrous THF was added and the sample was further sonicated for additional 10 min at 60 °C. Next, 1000 μL of Mill-Q at a rate of 8 mL/min was injected to the mixture while shaking the vial on an orbital shaker (1000 rpm), followed by sonication of the sample vials for another 5 min at 60 °C under a nitrogen environment (the sample was purged for 30 s with N_2_). Right after, 16 mL of acetonitrile at a flow rate addition of 5 mL/min was injected by means of a syringe pump (20 mm), while shaking the vial on an orbital shaker at a speed of 1000 rpm. Finally, the solution of beads was placed on a magnet (0.5 T). Next, the transparent supernatant was discarded and the dark pellet was dissolved in 5 mL of Milli-Q water. This magnetic washing was repeated three times to finally dissolve the pellet in 0.5 mL of Milli-Q water.

### Synthesis and Water Transfer of Iron Oxide Nanostars

IONS were prepared accordingly to our recently reported protocol.^23^ The IONCs were transferred from CHCl_3_ in water through a ligand exchange approach, using tetramethylammonium hydroxide (TMAOH) as ligand and following a slightly modified protocol reported in the literature.^24^ Briefly, 357 μL of IONS (8.4 mg_Fe_/mL, 13 ± 1) was precipitated by addition of 3 mL of acetone and centrifugation at 6000 rpm for 3 min. After discarding the supernatant, particles were redispersed in 1 mL of ethanol and sonicated for 10 min at room temperature. In a separate glass vial, 52 mg of TMAOH (550 ligands/nm^2^) was dissolved in 1 mL of ethanol. Then, the sonicated ethanol solution of particles was added dropwise into the ethanol/TMAOH mix and kept under sonication (60 Hz) for additional 30 min. Next, the TMAOH coated IONS (TMAOH-IONS) were washed on a cellulose membrane filter (Amicon Molecular Weight Cut Off, MWCO, of 100 kDa) three times by diluting the small sample volume (ca. 400 μL) on the filter each time with10 mL of Milli-Q water and finally concentrated the final sample to 400 μL in water.

### Synthesis of PMA-OD Premodified with 2,2′-(Ethylenedioxy)bis(ethylamine)

First, a functionalization of the PMA-OD with a primary diamine derivate, namely, (ethylenedioxy)bis(ethylamine), was performed according to a previous report.^9−11^ 350 mg (1 mmol in monomer concentration) of PMA-OD was dissolved in 10 mL of tetrahydrofuran (THF). 0.111 g of 2,2′-(ethylenedioxy)bis(ethylamine) (corresponding to 75% of the maleic anhydride monomer units) was added to the polymer solution. The solution was shaken at 60 °C for 5 h, concentrated to a final volume of 2 mL by applying a flow of nitrogen, and vortexed at 60 °C for at least 1 day. Subsequently, the solution was dried, and the product was redispersed in 20 mL of THF, followed by filtration using a 0.2 μm pore sized syringe filter. The as-obtained solutions were stored and used for the nanobead preparation. For the 50% and for the 25% functionalization, the protocol followed was exactly the same as described above for 75% with the only difference that a respectively reduced amount of 2,2′-(ethylenedioxy)bis(ethylamine) was used in the reaction instead of 0.111 g.

### Synthesis of Magnetic Nanobeads Prepared with a Premodified PMA-OD with 2,2′-(Ethylenedioxy)bis(ethylamine)

To perform a 1-fold nanobead synthesis, typically, 10 μL of Mn-IONPs with a mean diameter of 8.5 nm (concentration of iron and manganese ions 3.4 g/L and 0.5 g/L, respectively, from elemental analysis using ICP) toluene was dried under a flow of nitrogen, redissolved in THF, and mixed with a defined volume (30 μL of the 75% modified polymer solution) of the above-functionalized polymer solution). (It should be noted that we are not able to determine the nanobead concentration since we do not know exactly the number of nanoparticles per bead. Hence, we prefer to report the iron or manganese elemental concentration of the initial solution of magnetic beads.) The volume of the THF was chosen so that the total volume of the final polymer–nanoparticle solution was 200 μL. The mixture was shaken for 30 min at a rate of 1000 rpm. Subsequently, 0.8 mL of the destabilizing agent water was added to the mixture at a flow rate of 250 μL/min.

### Synthesis of Magnetic Nanobeads Prepared with a PMA-OD Premodified with *N*,*N*-dimethylethylenediamine for “Tertiary Amine Functionalization”

For comparison, a functionalization of the PMA-OD with a tertiary-amine derivate, namely, *N*,*N*-dimethylethylenediamine, was performed according to a previous report.^13^ 350 mg (1 mmol in monomer concentration) of PMA-OD was dissolved in 10 mL of tetrahydrofuran (THF). The respective amount of amino side chain molecules (corresponding to 75% of the maleic anhydride-*alt*-1-octadecene units) was added (0.0744 g of *N*,*N*-dimethylethylenediamine). The solution was shaken at 60 °C for 5 h, concentrated to a final volume of 2 mL by applying a flow of nitrogen, and vortexed at 60 °C for at least 1 day. Subsequently, the solution was dried, and the product was redispersed in 20 mL of THF, followed by filtration using a 0.2 μm pore sized syringe filter. The as-obtained solutions were stored and used for the nanobead preparation. Subsequently, the preparation of the nanobeads was following exactly the same procedure as described in the paragraph above.

### Synthesis of Magnetic Nanobeads Prepared with a PMA-OD Premodified with 2-(2-Pyridyl)ethylamine for “Pyridine Functionalization”

Similarly as described in the paragraph above, the polymer was premodified with 2-(2-pyridyl)ethylamine according to a previous report.^13^ Instead of 2-(2-pyridyl)ethylamine, here corresponding to 75% of the maleic anhydride-*alt*-1-octadecene units, 89 μL of 2-(2-pyridyl) ethylamine was inserted. After polymer prefunctionalization, the preparation of the nanobeads was following exactly the same procedure as described in the paragraph second above.

### Monoamino PEG750 Functionalization of Magnetic Nanobeads

In order to investigate the possibility to stabilize the colloidal solution in higher salt concentrations, a PEG-ylation of the nanobeads needed to be carried out. Therefore, a large sample was prepared by repeating 20 times the original above-described (1-fold) bead synthesis and cleaning it twice by magnet separation and redispersion in the same volume. The samples were combined and divided into two equal fractions. To one aliquot, 1 mmol of 1-ethyl-3-(3-(dimethylamino)propyl)carbodiimide (EDC) in 0.5 mL of borate buffer and 0.375 g of methoxypolyethylene glycol amine (aminoPEG750) in 0.5 mL of borate buffer were added, and the solution was vortexed for 2 h at room temperature. Both the aminoPEG750 modified and the nonmodified nanobead batches were washed four times by magnetic cleaning (0.3 T) and redispersing in 3 mL of Milli-Q water.

### Measurement Techniques

Dynamic light scattering (DLS) and the zeta potential (ZP) were recorded by means of a Malvern NanoZS instrument equipped with a 4.0 mW HeNe laser (633 nm). In order to measure the zeta potential versus pH dependency, each pH value was adjusted by adding the respectively necessary amount of hydrochloric or sodium hydroxide to a small amount of the nanobeads diluted with water (approximately 30 μL of the nanobead solution in 3 mL of total volume). For each of these measurements, a fresh aliquot of the nanobeads was employed.

Transmission electron microscopy (TEM) was measured on a JEOL JEM-1011 transmission electron microscope. The samples were prepared by drop casting a sample droplet onto a carbon coated copper grid followed by removing the liquid by evaporation under ambient conditions.

Fourier transform infrared (FTIR) spectroscopy was performed using a Vertex 70 V Bruker instrument having an attenuated total reflectance (ATR) configuration coupling a MIRacle ATR (PIKE Technologies). For the analysis, two polymer solutions at 50 mM (in monomer units) were prepared in THF or in 10% v/v water/THF mixture and the samples were shaken for 45 min prior to the measures. From these samples, 20 μL aliquots were drop casted on the zinc selenide crystal and allowed to dry before the measurement was done in vacuum.

AC hysteresis loops of magnetic samples made from IONS and their beads were measured using an AC magnetometer device (AC Hyster advance, Nanotech Solutions). Samples of desired concentration (2.3 mg_Fe_/mL diluted in water) were prepared in a capillar tube (diameter 3 mm, length 76 mm) and analyzed under dynamic magnetic field conditions of frequency 110 kHz and field range 16–24 kA/m.

The concentrations of the nanoparticles were determined by means of an inductively coupled plasma atomic emission spectrometer (ICP-AES, iCAP6500, Thermo). Therefore, typically, the samples were dried, dissolved in aqua regia, and subsequently diluted with a defined amount of pure Milli-Q water. Nuclear magnetic resonance (NMR) spectra were recorded on a BRUKER DRX-400 spectrometer. Deuterated chloroform and methanol were used and indicated in brackets for each compound. Chemical shift values (δ) are referred to tetramethylsilane used as internal reference.

## 3 Results and Discussion

The simple method we previously proposed for the assembly of magnetic nanoparticles in nanobeads was based on the coprecipitation of the IONPs and an amphiphilic polymer occurring by a process of destabilization of both components promoted by a solvent at a polarity different from that of the solvent in which the NPs and the polymer were initially dissolved.^7^ More specifically, a solution of IONPs and polymer poly(maleic-*alt*-1,1-octadecene) (PMA-OD) was dissolved in tetrahydrofuran (THF), and after shaking this solution for 45 min, a more polar solvent, acting as antisolvent, was added dropwise to induce NPs and polymer precipitation. As the preferred antisolvent, acetonitrile (ACN) was often chosen. During the addition of ACN, the polymer–NP solution starts to precipitate by forming polymer beads encapsulating the hydrophobic magnetic NPs, coated by alkyl surfactants, in the hydrophobic core of the polymer beads. This process depends on the difference in polarity of the two solvents and on the rate by which the nonsolvent was usually added. However, the synthetic route turned out to be furthermore sensitive to a large number of parameters. In particular, the ratio of the amounts of IONPs to polymer, the time and the speed of shaking, the water molecule/monomer unit ratio, the choice of solvents and the solvent ratio, and the humidity of the air can all affect the nanobead quality. By optimizing all these parameters, we found a recipe to get very regular nanobeads, which were monodisperse in size, had a reasonable polymer shell thickness and contained a sufficient amount of magnetic NPs (or even more than a type of NPs) per beads to promote their fast magnetophoresis mobility.^6,17,19^

Here, in a set of experiments performed with Mn-IONPs and PMA-OD polymer, it was observed that changing the stirring time in THF and the amount of THF used as solvent and performing the bead synthesis in closed or open vials led to nanobeads of different quality (data not shown). This observation alerts us to the importance of the effect of humidity (water) on the bead protocol, since ring opening of the maleic anhydride polymer plays an important role in the solubility of the reaction solvent and in turning on the bead formation. To verify the ring opening of the maleic anhydride groups after addition of water to the polymer in THF, FT-IR characterization was performed and compared to the same polymer in THF (with no water addition). Upon water addition followed by shaking for 45 min, peak shoulders appeared at 3210 cm^–1^ and 835 cm^–1^ in this sample (Figure S1, red plot), which correspond to the stretching and bending vibrational modes, respectively, of the O–H groups of the carboxylic acid, thus providing the indication of the ring opening of the maleic anhydride groups.

**Figure 1:**
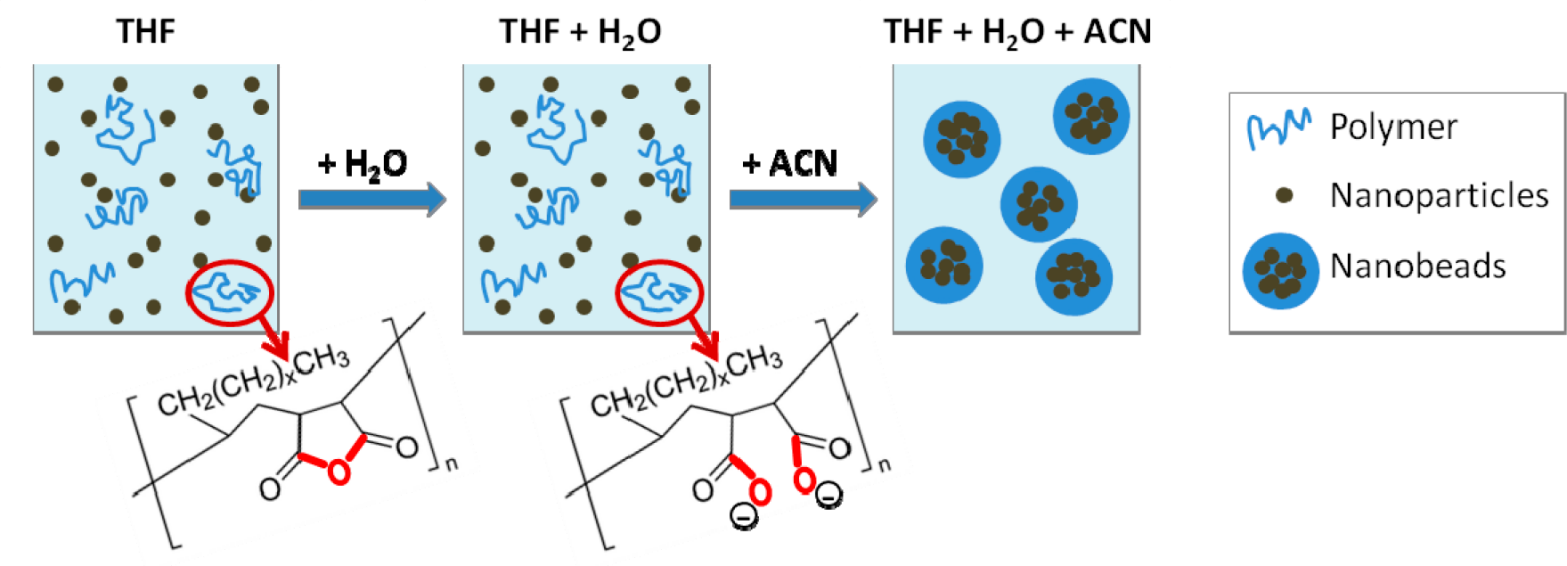
Schematic drawing of the nanobead formation in the presence of water. Initially (left), when dissolving the nanoparticles with the polymer, all maleic anhydride rings are closed. After adding a controlled amount of water and vortexing for a certain time (center), some of the maleic anhydride rings are opened while both the polymer and the nanoparticles remain in solution. Opening of the maleic anhydride of this polymer to a certain degree affects the solubility in more polar solvents, and on the other hand, the stability of the colloidal nanobeads formed owing to their resulting surface charge and hence electrostatic repulsion. Therefore, the nanobeads formed during the addition of acetonitrile (right) are colloidally stable in solution.

To report about a more reproducible and scalable protocol, the bead protocol was modified such that it did not depend on the humidity parameter in an uncontrolled manner. Indeed, it was expected that by controlling very carefully the amount of water added in the reaction mixture during the nanobead synthesis, the hydrolysis of the anhydride groups would occur much more reproducibly, providing changes in polymer solubility, thus, in turn, affecting the polymer/NP assembly (Figure 1). Therefore, to fully control the polymer hydrolysis, we conducted the nanobead synthesis under nitrogen conditions, using anhydrous solvents and at gradually increased amount of water added to the vial together with Mn-IONPs and polymer while keeping all the other reaction parameters (*i.e.*, polymer amount, NP amount, solvent addition rate, shaking time) the same as set for what we call “the standard synthesis of nanobeads” protocol (see Materials and Methods section for a full detailed protocol).

**Figure 2:**
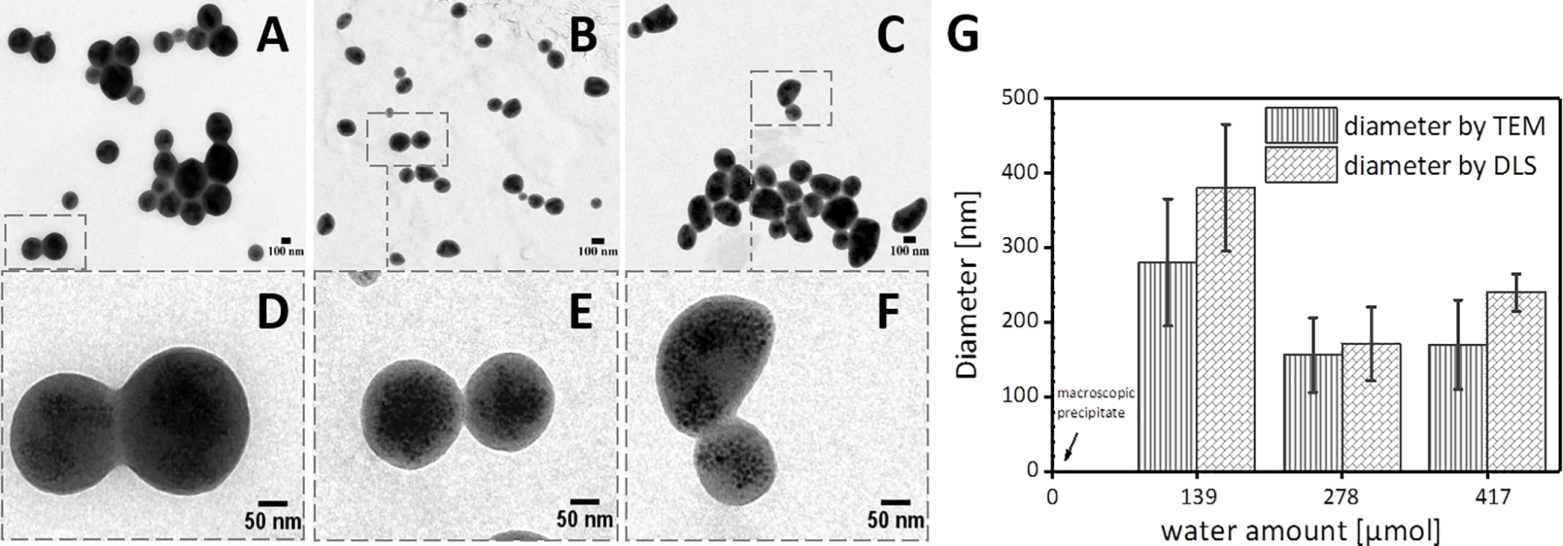
TEM images of nanobead synthesis under controlled water amount addition of (A) 2.5 μL (139 μmol), (B) 5 μL (278 μmol), and (C) 7.5 μL (417 μmol) of water added to the anhydrous reactants (polymer and Mn-IONPs in THF) followed by the addition of acetonitrile solvent. (Zoom D–F) Higher magnification of TEM image of the nanobeads. (G) Analysis of the nanobead diameters by TEM and by DLS as a function of the water amount added. The error bar indicates the standard deviation of the size distribution by TEM analysis on at least 200 nanobeads analyzed and the standard deviation of the DLS analysis from three independent measurements.

It was observed that when working in an anhydrous environment and with no addition of water, macroscopic agglomeration occurred. Instead, by addition of defined amounts of water at volume of 2.5 μL, 5 μL, or 7.5 μL under the same bead conditions as for the sample with no water, nanobeads were formed in all cases (Figure 2A–C, respectively). Note that, for water volumes of 2.5 μL, 5 μL, and 7.5 μL, the corresponding micromole amounts of water added were respectively 139 μmol, 278 μmol, and 417 μmol. Magnified TEM images of the nanobead obtained for each water amount are shown in Figure 2D–F, and by performing TEM analysis of the nanobeads size, it is found that the average diameter of the beads decreased with increasing water amount added from 280 ± 85 nm to 170 ± 60 nm. This result suggests that a minimum amount of water was needed to possibly hydrolyze the polymer backbone and hence to increase its solubility in the reaction mixture. The same trend was observed by dynamic light scattering (DLS) analysis when analyzing the hydrodynamic diameter (*d*_H_) for the same set of samples which was found to vary in the range from 380 ± 85 nm to 240 ± 25 nm when the water amount was increased (Figure 2G). The larger *d*_H_ of the beads in DLS with respect to the TEM diameter was expected given the hydration of the charged polymer at the bead surface in aqueous solutions. Moreover, the statistical analysis by TEM and DLS of the beads size indicates that it is possible to synthesize magnetic nanobeads with a reasonable size distribution (Figure 2). Visual inspection on several beads by TEM analysis also suggests the presence of reasonable magnetic NP content for all the samples at different water contents (Figure 2). Note that the nanobeads produced with only a small amount of water (2.5 μL) were significantly larger and more polydisperse than those produced with more water added. Indeed, for these larger nanobeads the standard deviations measured by TEM and by DLS were the highest (almost twice as big) compared with standard deviations for the other two batches prepared in the presence of more water (Figure 2). The polydispersity indexes (PDI) as obtained from DLS measurements were 0.11, 0.053, and 0.056, respectively, supporting the more heterogeneous size for the sample prepared with less water than for two samples prepared with more water. These observations indicate that the amount of water added to the polymer NP mixture significantly affects the quality of the resulting nanobeads.

Bearing in mind these results obtained on the small 1-fold (1×) batch in the 4 mL vials, next, an attempt to scale up this procedure was made. The same synthesis was indeed carried out in a 20 mL vial, still under a nitrogen atmosphere, tuning the volume of water, polymer, and Mn-IONPs added into the vial by well-defined scale-up factors with respect to the 1-fold nanobead reaction. Indeed, with this route, we successfully performed scale-up syntheses using 20, 40, 80, or 100-fold the amounts (in volume) of water, NPs, and polymer. For these scale-up reactions, the volume of THF added was adjusted so that the total volume of the reaction (THF, volume of PMA-OD, volume of water, and Mn-IONPs) was kept below 6 mL while the amount of ACN was always fixed to 16 mL so that the total volume of the product solution was always below 20 mL. This means that the solvent and destabilizer solvent amounts were not scaled up accordingly to the scale-up factor chosen in the 1× protocol. This choice was required in order to handle a fairly large volume of the beads solution to allow the overnight magnetic separation of the formed magnetic beads from the supernatant when applying an external magnet (0.3 T).

**Figure 3:**
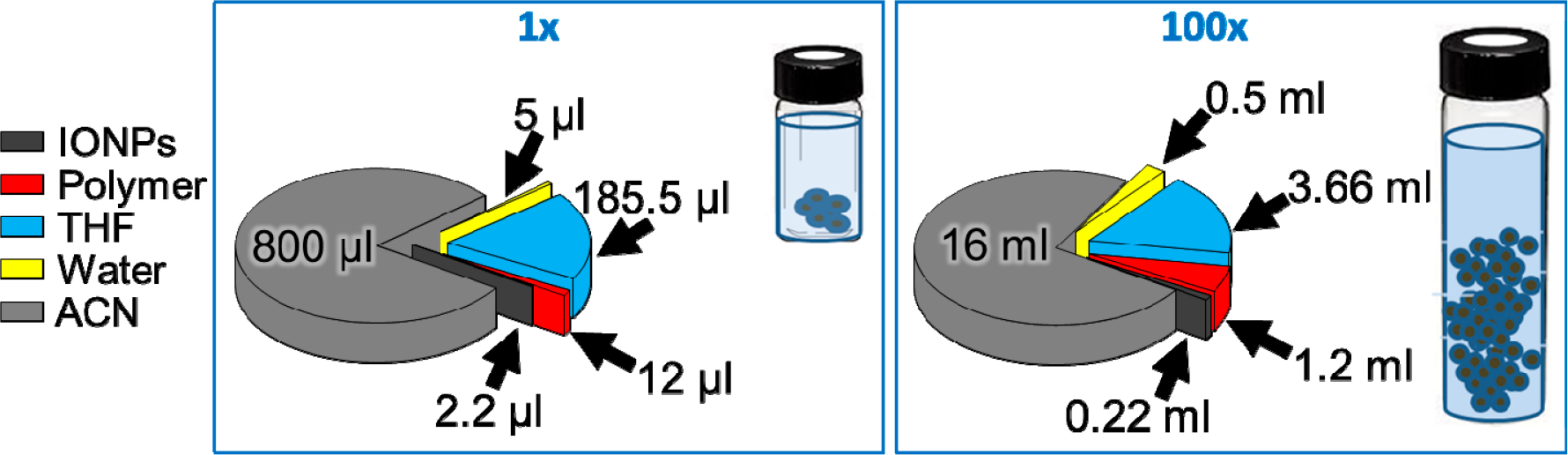
Modification of the upscale procedure from the water-controlled nanobead synthesis (left) and a 100-fold upscale procedure (right). The amounts of water, IONPs, and polymer solution were increased by 100-fold, while the amount of THF added to the polymer+IONPs+water solution was adjusted to reach a volume below 6 mL and the ACN solvent volume was fixed to 16 mL. Note that the volume of Mn-IONPs is always evaporated before addition of polymer solution, water, and THF.

Figure 3 reports a practical example of scaling up of nanobeads for the case of a 100-fold increase of chemicals with respect to the small batch (1× batch) for the case of addition of 5 μL water in the 1× batch: while the volume of water, polymer, and Mn-IONPs was scaled by a factor of 100 with respect to the small batch, the volume of THF was adjusted to have a total initial reaction volume of below 6 mL considering the volume of the polymer+IONPs+water added. The volume of ACN was always fixed at 16 mL for the 20×, 40×, 80×, and 100× protocol. It is worth noting that the water volume was added to the mixture at a flow rate of 8 mL/min and the ACN was added within the same time span (200 s) as it was in the standard (nonupscaled) experiments.

**Figure 4:**
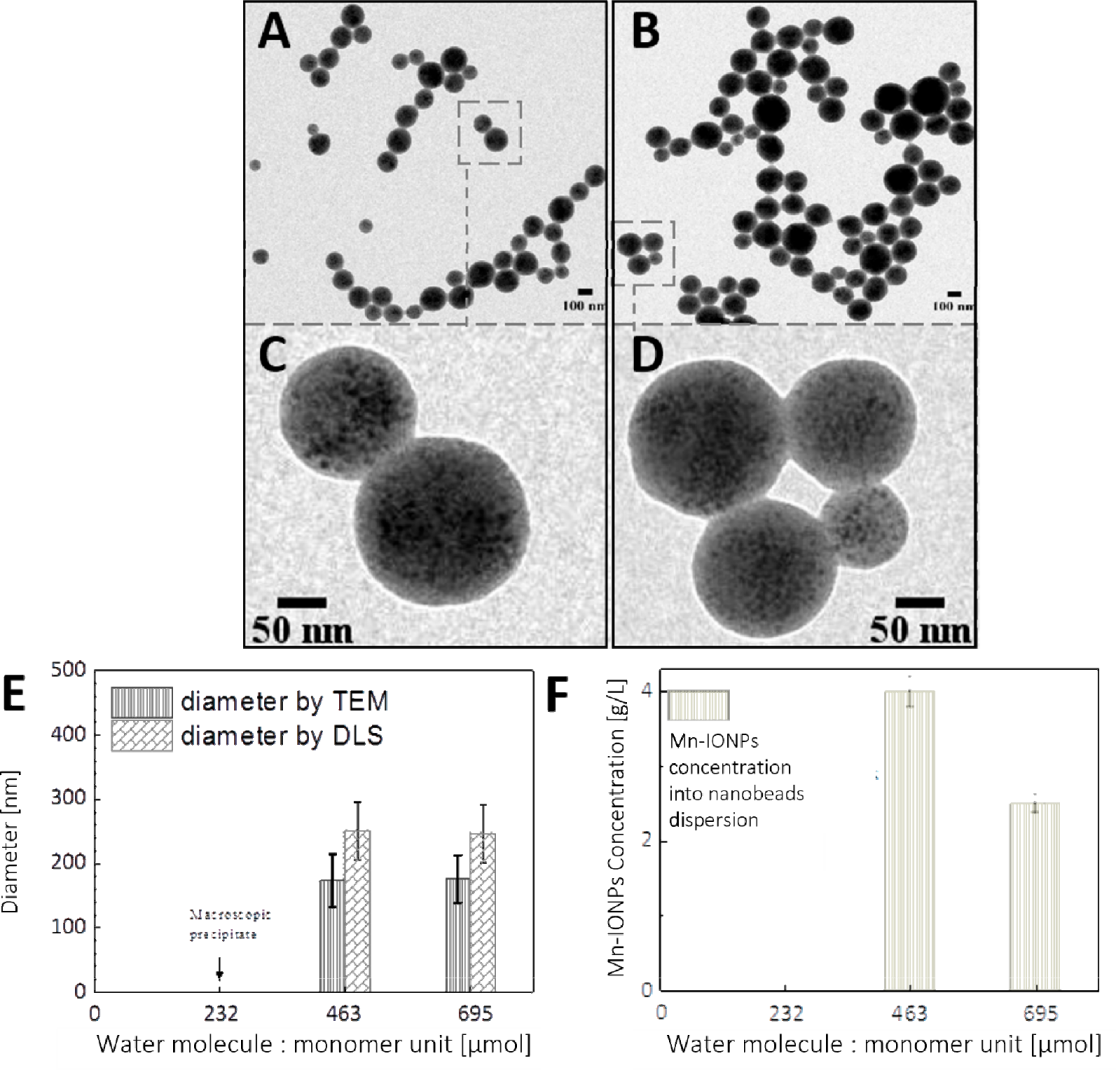
TEM image (A,B) and higher magnification TEM image (zoom C and D) of nanobeads produced by a 100 times scale-up approach and at a water content of 500 μL (A) and 750 μL (B). (E) Analysis of the nanobead diameter by TEM (left columns) and by DLS (right columns) as a function of the water/polymer ratio. The error bar indicates the standard deviation from the size distribution by TEM and DLS analysis measured by three independent measurements. For those two nanobead samples, the PDIs as obtained from DLS measurements were 0.063 and 0.077, respectively. (F) Concentration of l Mn-IONPs measured on the nanobeads dispersion.

In the next set of experiments, to evaluate the effect of water on the beads formation for the 100× batch, the amount of water volume was systematically varied from 0 μL to 750 μL, while the volume amount of Mn-IONPs and polymer was kept at 100-fold, as well as that of THF and ACN fixed at the indicated values described above. The analysis of a typical 100-fold scaled up nanobead synthesis, which was performed by controlling the amount of water (0 μL, 250 μL, 500 μL, and 750 μL of water), is shown in Figure 4. The samples synthesized in the absence of water and with the smallest amount of water (250 μL) resulted in macroscopic aggregation visible under eye inspection. The samples synthesized with water volumes at 500 μL (which correspond to a water/monomer unit of polymer ratio of 463) and at 750 μL (water/monomer unit of polymer ratio of 695) resulted in well-soluble brown solutions showing well-defined nanobeads as under TEM analysis (Figure 4A,B, respectively). Note that, in each sample, to 220 μL of nanoparticles (80 μM Mn-IONPs, 8.5 nm in diameter) after solvent evaporation under a nitrogen flow were added 1200 μL of an anhydrous PMA-OD polymer solution (50 μM in monomer units) and 3.660 mL of anhydrous THF for the sample reported in Figure 4A or 3.610 mL for the sample reported in Figure 4B, respectively. Next, a volume of deionized water was added to each vial at a rate of 8 mL/min followed by 45 min of vortexing at 1000 rpm. Final addition of 16 mL ACN was added at a rate of 5 mL/min. In Figure 4E, the nanobead mean sizes estimated by TEM statistical analysis and DLS measurements are shown with a zoom of the nanobeads shown in Figure 4C,D. The TEM diameters were in the range 130 to 200 nm, while DLS analysis reveals sizes that are approximately 70 to 150 nm larger (see Figure 4). This trend was also shown for nanobead synthesis scaled at 20, 40, and 80 times factor (Figure S2). The analysis by TEM and DLS indicates that also in this case, by controlling the total water content, it was possible to synthesize magnetic nanobeads with a regular spherical shape, reasonable size distribution (polydispersity index from DLS being of 0.063 and 0.077 for the two different batches, respectively), and reasonable NP content (Figure 4). From the higher magnified TEM image it can be observed that the magnetic NPs were also located inside the nanobeads. Mn-IONP amounts for these bead solutions were also determined by iron elemental analysis performed by ICP, confirming a quantitative amount of iron (thus Mn-IONPs) in the bead solutions (Figure 4F). Note that, for these two samples at water contents 500 μL and 750 μL, in any of the preparation steps of the bead reaction no loss of magnetic NPs was observed (no brown residuals were left in the glass vials or in the filter during purification). This visual observation was also confirmed by ICP elemental analysis measurements of the iron associated with the nanobead solutions prepared by 100-fold methods for those two samples at different water contents. The recovery yield of Mn-IONPs by iron ICP analysis in the magnetic bead fraction was nearly 100% with respect to the nominal amount of iron found in the reaction mixture. This suggests that no significant loss of magnetic material was observed during the bead formation.

From our findings we can conclude that, even though the added water varies for different batch sizes, working under conditions with a controlled value of the water molecules/monomer units ratio leads to a more reproducible nanobead synthesis. Furthermore, the amount of water is the key for scaling up the nanobead synthesis.

After synthesis, the nanobeads form a colloid solution, which is stable for several days before settling down by gravity. The nanobeads, however, can be easily redissolved in solution just by shaking the sample vial.

**Figure 5:**
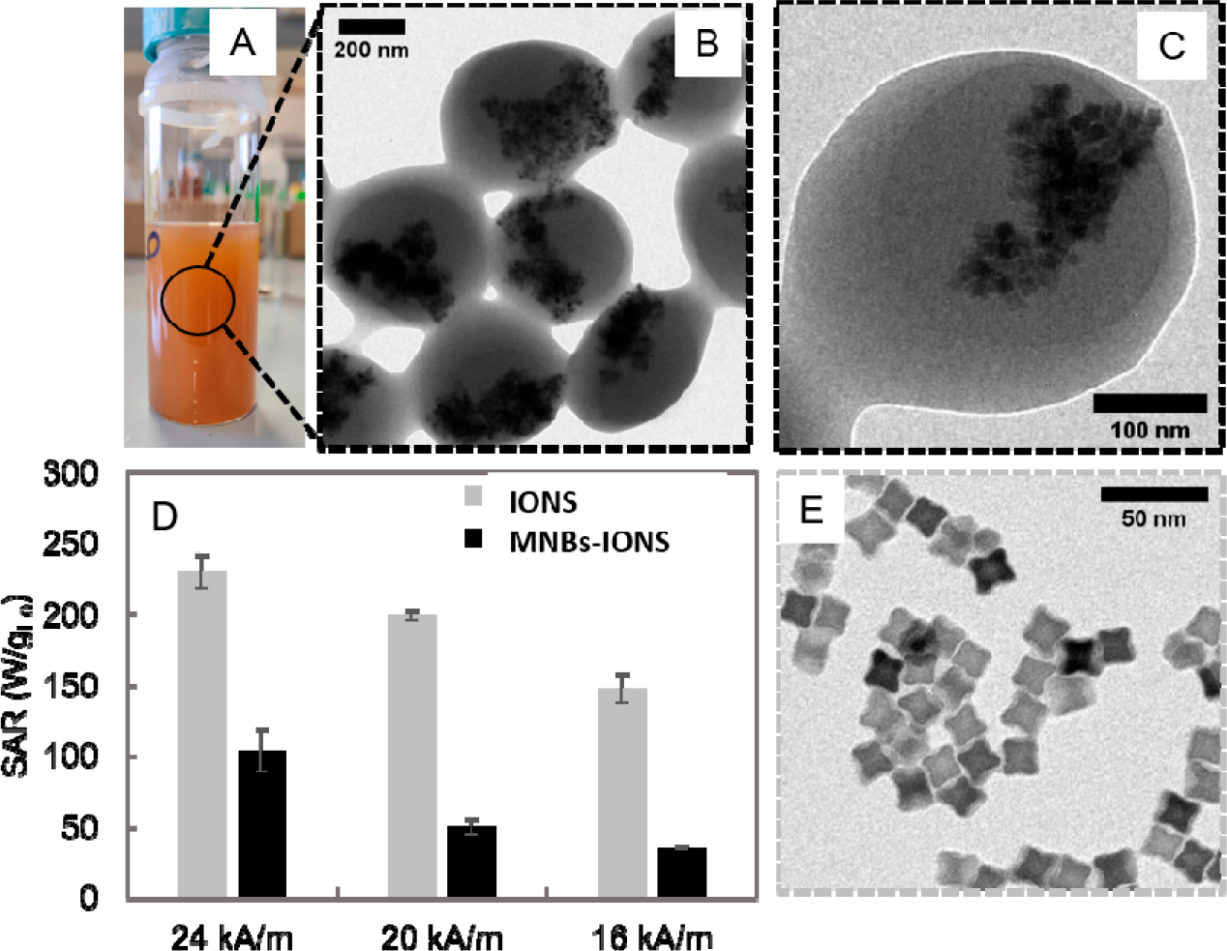
Implementation of the nanobeads scale-up protocol to another type of magnetic nanoparticles, the IONS with a star shape and a size of 13 ± 1 nm. (A) Photograph of magnetic nanobeads (MNB-IONS) solution. (B, C) TEM images of the magnetic nanobeads at low and high magnification. (E) TEM image of the individually coated IONS stabilized in water with TMAOH. (D) SAR values of MNBs-IONS and IONS solutions based on the hysteresis loops measured by AC magnetometry at frequency of 110 kHz and field range of 16–24 kA/m at a fixed concentration of 1 g_Fe_/L in water.

The scale-up protocol (100-fold reaction) was also implemented on other magnetic nanoparticles, which present a peculiar star-shape and are made of iron oxide, the IONS. These nanoparticles produced through a solvothermal method, thanks to their anisotropic shape, possess the ability to convert magneto-energy into heat with a high efficiency when exposed to an alternating magnetic field of clinical use.^1,15^ The protocol steps for the production of IONS based beads were mostly the same as for Mn-IONPs based beads but some modifications were required (see Table S1 for the direct comparison of the parameters). In particular, all the shaking steps were replaced with the more energetic sonication steps, helping to better disperse the nanoparticles in solution at each of the production steps. Also, prior to the addition of polymer, an extra amount of OA, the surfactant that stabilizes the IONS nanoparticles surface in THF, was required. Further, the protocol for IONS was set with an amount of nanoparticles that was less than the one employed for Mn-IONP beads (roughly one-third in metal content was used for the IONS beads). Importantly, the volume of water added to favor the opening of the anhydride groups was more than the one used in the synthesis of Mn-IONPs beads, but it was kept under sonication for a short time, prior to the addition of ACN solvent. To briefly summarize the protocol, the IONS were sonicated in the presence of oleic acid prior to CHCl_3_ solvent evaporation. Next, upon solubilization of IONS with PMA-OD polymer in anhydrous THF, water was added and the mixture was sonicated at 60 °C. The beads solution formed (Figure 5A) after addition of ACN. Finally, the IONS-MNBs sample was magnetically washed three times (see Materials and Methods for details). Note that all these proposed changes were needed because, if the standard 100-fold protocol of Mn-IONP-MNBs was applied to the IONS, the magnetic materials precipitated out of the milky polymer solution (Figure S3).

The morphology of magnetic nanobeads by TEM analysis indicates the formation of beads (MNB-IONS) with a clear polymer shell and randomly assembled IONS in the core (Figure 5B and Figure S3C). DLS analysis reveals IONS-MNBs with hydrodynamic curves with monomodal distribution (Figure S4). Hysteresis loops at 110 kHz of MNB-IONS measured with a AC magnetometer (Figure S5) were recorded to extract the specific adsorption rate (SAR) values according to the formula SAR = *A* × *f*/*m*_Fe_, where *A* is the area of the hysteresis loops recorded at the frequency (*f*) of measure and normalized to the iron mass (*m*_Fe_).^25^ SAR values comparison suggests that the SAR values of MNBs-IONS are lower at all field conditions tested than that of IONS stabilizes in water as single nanoparticles, but the values are still significant considering that they have been measured at a clinically used frequency of 110 kHz (Figure 5B,C). Indeed, the SAR values for those MNBs are similar to the one reported for iron oxide nanoparticles used in clinical trials.^26^ The lowering of the SAR values for the magnetic nanoparticles encapsulated in a polymer beads are expected indeed, as unfavorable magnetic dipolar coupling interactions occurs for nanoparticles packed in a centro-symmetrical structure. Moreover, the overall size of the beads is increased with respect to the individual nanoparticles, which in turn, affects the Brownian relaxation time of the magnetic materials with a reduction of the heating efficiency.

**Figure 6:**
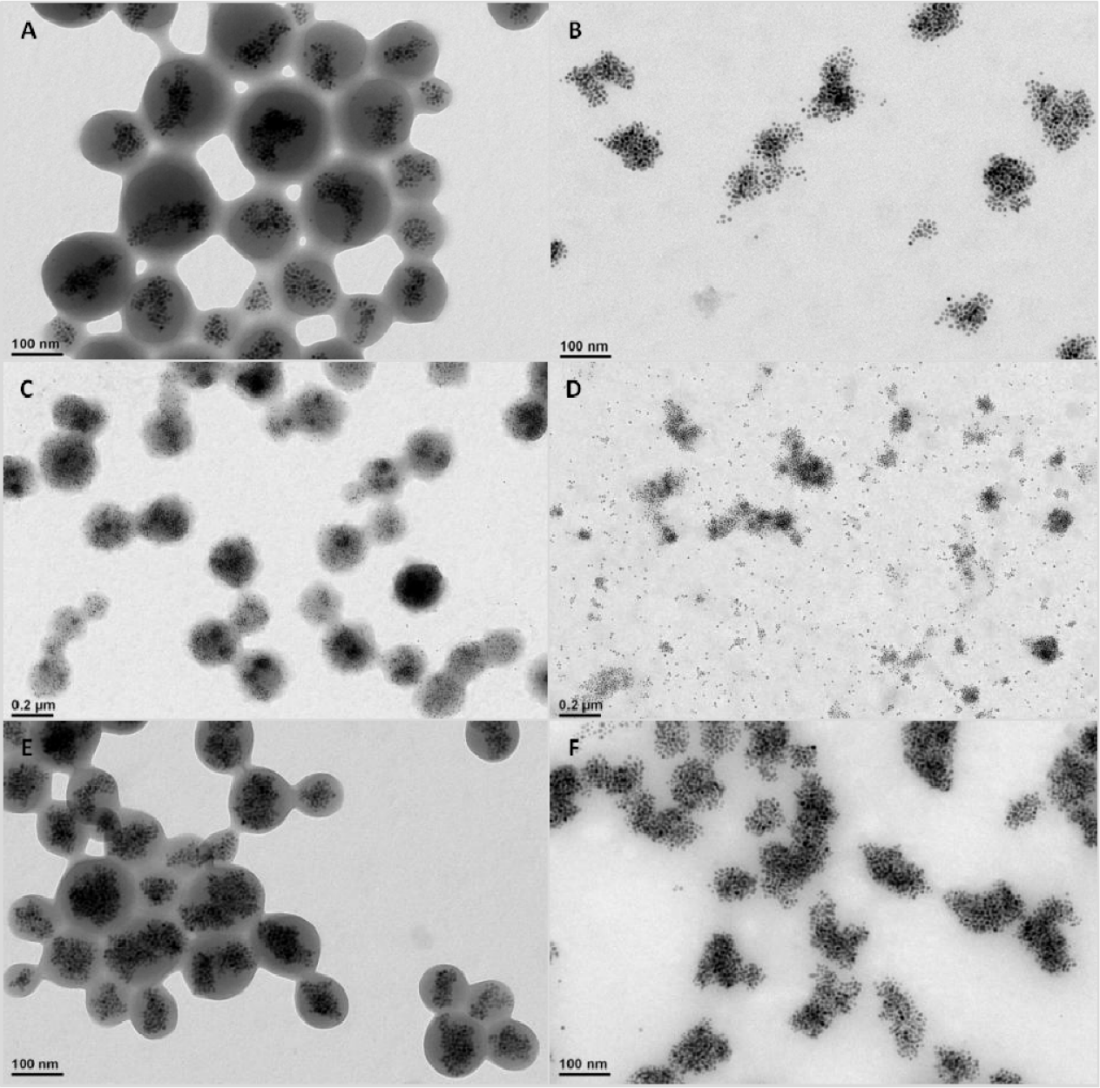
Overview of the TEM image of magnetic nanobeads obtained from PMA-OD (A) before and (B) after modification with amino-PEG750 *via* EDC chemistry. (C) and (D) are TEM images of magnetic beads from PMA-OD prefunctionalized with a tertiary amine (*N*,*N*-dimethylethylenediamine), before and after amino-PEG750, respectively. (E) and (F) are TEM images of beads functionalized with an amino derivate of a pyridine (2-(2-pyridyl)ethylamine), before and after PEG-ylation. In all three cases, after the reaction of the shell with amino-PEG750, the polymer beads are not visible anymore and only groups of nanoparticles can be distinguished, indicating a loss of polymer structures around each bead due to PEG functionalization reaction.

**Figure 7:**
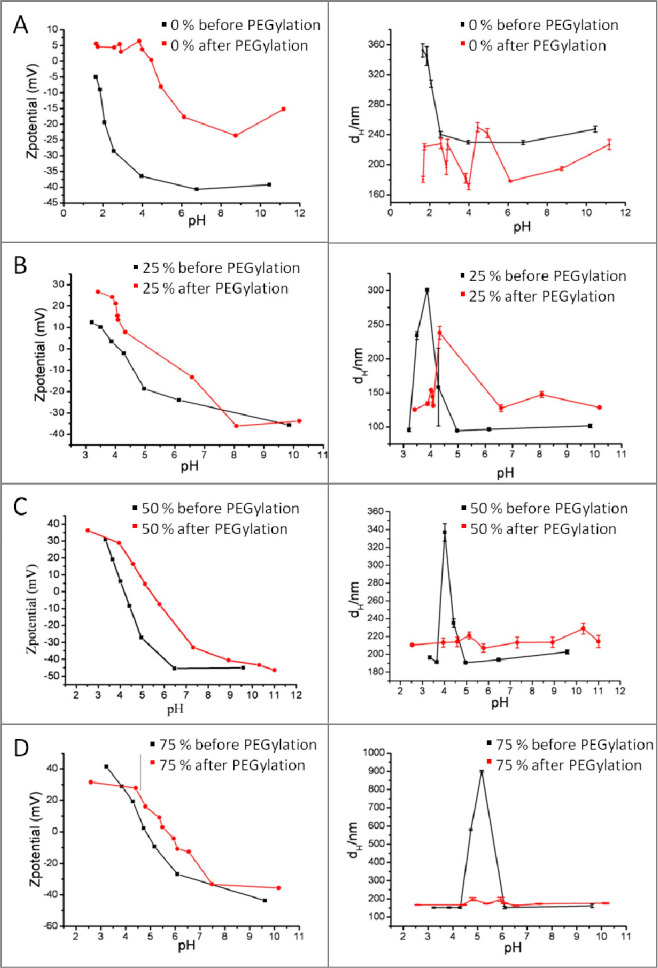
(left) Zeta potential and (right) hydrodynamic diameter (*d*_H_) from the DLS curve as a function of the pH before and after monoamino PEG750 reaction of the solution for (A) nanobeads made from PMA-OD and (B–D) nanobeads made from PMA-OD prefunctionalized with primary amine side chain at 25%, 50%, and 75% of the polymer monomer units, respectively. Even though the nanobeads sizes are not discrete anymore after PEG-ylation, the colloidal stability of the beads seems to remain, and the net zeta potential has increased.

Generally, in previously reported routes, upon changing the pH or upon exposing the nanobeads to higher ionic strength media, destabilization of the nanobead colloid was observed. This is likely because the carboxylic groups on the polymer on the nanobeads can undergo protonation and screen charge effects in the case of change in pH (acidification) or in the presence of salt species. To stabilize the magnetic nanobeads by steric repulsion rather than just negative charge repulsion and hence increase the colloidal stability of nanobeads, a post amino-PEG functionalization reaction is usually carried out by standard EDC chemistry on the as-prepared nanobeads with amino-PEG750. In this case the PEG molecules work as pillow molecules and the nanobeads are less affected by surface charge changes.^9^ As shown by TEM characterization, it turned out that, upon monoamino-PEG-ylation, the polymeric nanobead structures were either partly dissolved or swollen; hence, the polymer bead edges appeared less defined than before functionalization (Figure 6). The DLS data of these PEG-ylated nanobeads confirmed less appearance of agglomeration with respect to the nonpegylated nanobeads, but still large oscillations in *d*_H_ as a function of the pH were measured (Figure 7A,B). To maintain a more stable polymer structure around the magnetic NPs, a further modification of the nanobead synthesis was developed, which allows us to introduce a cross-linking agent on the polymer to be used for the nanobead preparation. More properly, before the bead synthesis, we premodified the PMA-OD polymer by reacting some of the anhydride groups with short molecules bearing diamino terminated moieties, namely, 2,2′-(ethylenedioxy)bis(ethylamine).^9^ It is worthwhile to note the amide functionalization of the PMA-OD polymer with the 2,2′-(ethylenedioxy)bis(ethylamine) at different percentages was confirmed by proton NMR characterization (Figure S6). The amine groups on these molecules would react toward the anhydride groups of the polymer, during nanobead formation, to form amide bonds, thus promoting the cross-linking of the polymer shell and providing a more compacted polymer shell at the nanobeads surface.

Controlling the ratio of diamino terminated molecules per polymer chain would enable control of the cross-linking during the bead formation. Figure 7 shows the TEM characterization of nanobeads before and after the prefunctionalization of the polymer with the diamino-PEG-ylation with different amounts (25%, 50%, and 75%) of primary amine side chains added. Contrary to nanobeads made from the pristine PMA-OD as well as for those prepared from the same polymer premodified with amino tertiary amine derivates (*N*,*N*-dimethylethylenediamine) or amino pyridine derivates (2-(2-pyridyl)ethylamine) (as reported in Bigall *et al*.^13^) and therefore missing the possibility to cross-link the polymer shell (see Figure 6 and Figures S7 and S8), nanobeads prepared with polymer prefunctionalized with primary amines did not exhibit a decreased contrast in electron microscopy (Figure 7). This effect is attributed to the fact that free amino groups of the diamino molecules attached to the polymer chain by cross-linking the polymer shell reacting to the anhydride groups during bead formation maintain a tide polymer shell even during surface functionalization of the beads.

**Figure 8:**
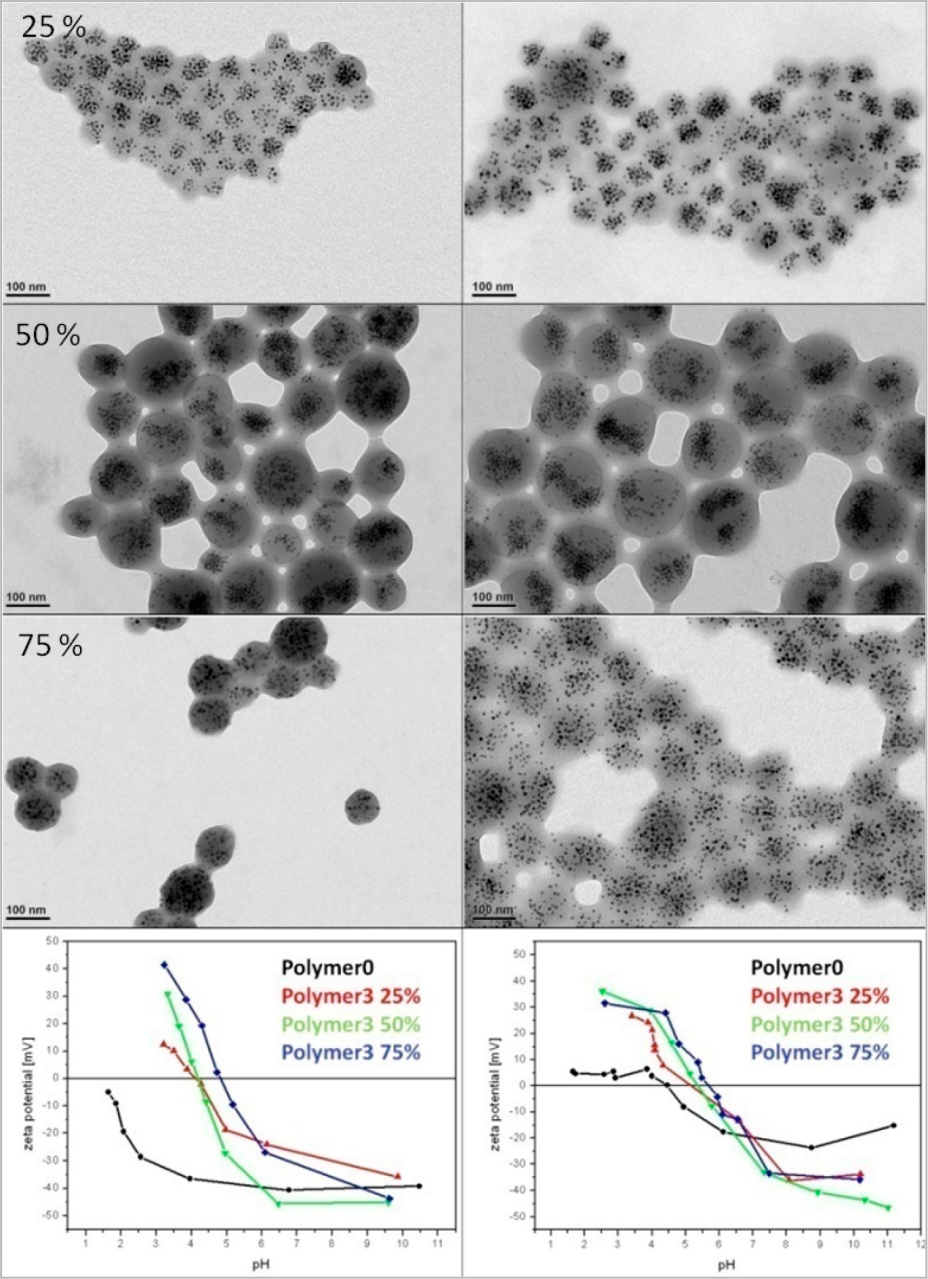
(Left column) TEM images of nanobeads prepared from diamine (2,2′-ethylendedioxy)bis(ethylamine) functionalized polymers with 25%, 50%, and 75% diamine molecules added with respect to the maleic anhydride monomer groups. (Right column) TEM images of the same nanobeads after functionalization with poly(ethylene glycol). In the bottom line, the pH dependency of the differently modified polymer nanobeads is shown (left) before and (right) after the monoamino-PEG750 functionalization. Key: polymer0, no amine functionalization; polymer3, functionalization with 25, 50, and 75% of the diamine molecules added with respect to the maleic anhydride groups.

It can be observed that for polymer functionalization larger than 25%, after monoamino-PEG750 functionalization no agglomeration appears in pH regimes close to the isoelectric point. Generally, the zeta potential (pH dependency shown in Figure 8) turns out slightly more positive after the PEG functionalization, which is in good agreement with the idea of linking amino-PEG molecules to existing carboxy groups of the polymer and hence reducing the amount of carboxyl groups expressed on the nanobead surface.

Therefore, we deduce that the nanobeads can be better stabilized by modification of the commercial PMA-OD polymer with primary diamine molecules prior to the nanobead formation and that subsequent PEG-ylation of the respective nanobeads can work without much effect on the nanobead structures. This functionalization may become very important when magnetic-fluorescent nanobeads made of two different types of NPs, such as quantum dots and iron oxide NPs, are employed. Indeed, as previously shown by Di Corato *et al*.,^9^ while the magnetic NPs are included more in the core of the beads, the quantum dots are distributed more into the polymer shell. As shown here, a functionalization of the nanobeads on a non-cross-linked (thus unstable) polymer shell may expose the fluorescent quantum dots to the solution environment with problems of fluorescent stability. On the contrary, a prefunctionalized diamino side chain polymer with cross-linking of the shell would allow for the maintenance of a tidy shell, as shown here, thus allowing further process of the bead surface without potentially compromising the fluorescent properties of the beads.

## 4 Conclusions

We have developed a scale-up approach for the synthesis of colloidal nanobeads from magnetic nanoparticles and poly(maleic anhydride-*alt*-1-octadecene) polymer. Our suggested route can work with different types of nanoparticles. Here, for the sake of simplicity we chose two types of superparamagnetic nanoparticles, namely, manganese ferrite and iron oxide nanostars. Manganese ferrite nanoparticles are of interest as MRI contrast agents, while iron oxide nanostars are exploitable as heat mediators in the so-called magnetic hyperthermia treatment thanks to their ability to covert magneto-energy into heat upon exposure to alternating magnetic field of clinical used.^11,23^

The reproducibility of the nanobeads was improved by controlling the amount of H_2_O content present in the reaction mixture. With such control of the water content, it was also possible to obtain in one single shot, high-quality nanobeads at a scale-up factor of 100 times, enabling us to produce a nominal amount of nanobeads at 7 mg in iron with respect to the 70 μg of the 1-fold. Premodification of the polymer prior to the bead synthesis with primary amine molecules, like the diamine 2,2′-(ethylenedioxy)bis(ethylamine), used here as a side chain leads to a cross-linking and very compact polymer shell, which prevents the polymer beads from swelling or dissolving upon further functionalization or manipulation of the shell at different pHs. These findings display significant steps toward the application of such magnetic nanobeads in biomedical imaging, hyperthermia, or magnetically guided drug delivery, for future *in vivo* experiments where large amounts of stable nanobeads will be required.

## Supporting Information Available

FT-IR spectra, TEM and DLS diameter analysis of nanobeads, TEM images and solution photograph, hydrodynamic size spectra and table of hydrodynamic sizes, AC hysteresis loops, ^1^H 400 MHz NMR spectra in DMF-*d*_7_ of side chain molecules attached to the PMA-OD, and summary of the reaction conditions (PDF)
