# Supplementary material for: Scaling Up Magnetic Nanobead Synthesis with Improved Stability for Biomedical Applications
Source: J Phys Chem A. 2022 Dec 16;126(51):9605–17. doi: 10.1021/acs.jpca.2c05902 (PMC9806829; doi:10.1021/acs.jpca.2c05902)
Supplement: Supplementary file 1 — jp2c05902_si_001.pdf [file jp2c05902_si_001.pdf]

Supporting Information

Scaling up Magnetic Nanobead Synthesis with Improved Stability for Biomedical Applications

Nadja C. Bigall[1,2,†], Marina Rodio[1 †], Sahitya Avugadda,[1†] Manuel Pernia Leal[1,3], Riccardo Di Corato[1,4], John S. Conteh,[1] Romuald Intartaglia[1], Teresa Pellegrino[1*]

[1] Istituto Italiano di Tecnologia, Via Morego 30, 16163 Genova, Italy

[2] Leibniz Universität Hannover, Callinstr. 3A, 30167 Hannover, Germany

[3] Universidad de Sevilla, Facultad de Farmacia, Departamento de Química Orgánica y Farmacéutica, c/ Profesor García González, 2, 41012 Sevilla, Spain.

[4] CNR, Institute for Microelectronics and Microsystems (IMM), Via Monteroni, Lecce 73100, Italy

† These authors contributed equally to this work.

*E-mail: teresa.pellegrino@iit.it

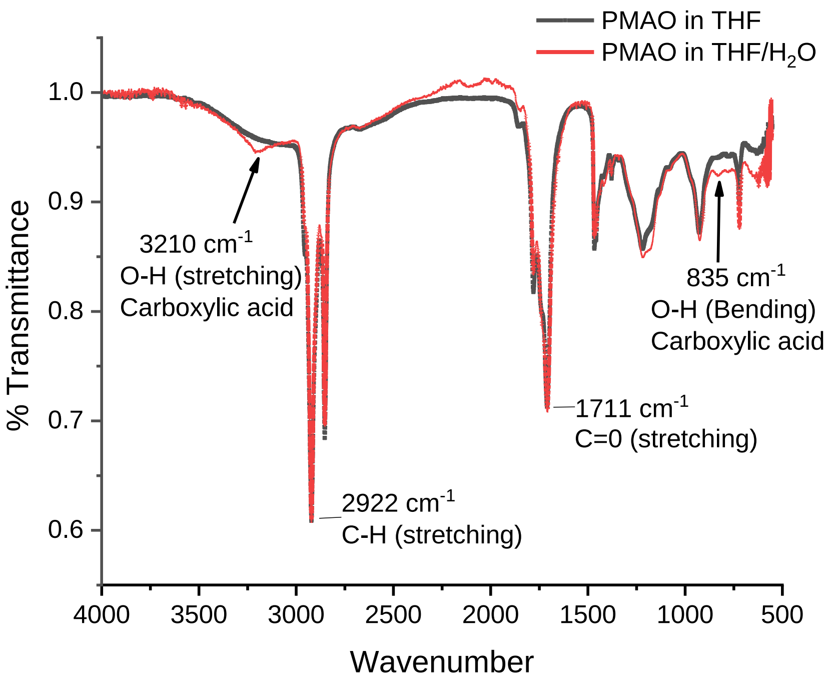

Figure S1. *Comparative FT-IR spectra of poly(maleic anhydride-alt-octadecene) PMAO in THF (black curve) and PMAO in a 10% v/v water/THF mixture (red curve), confirming the successful opening of the maleic anhydride ring after water addition.*

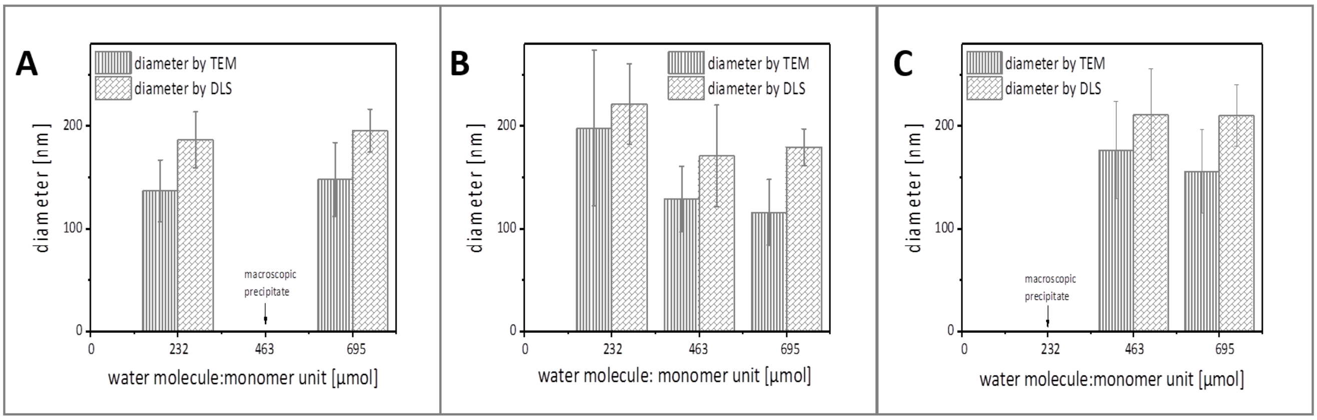

Figure S2: *TEM and DLS diameters of nanobeads prepared from an (a) 20-fold, (b) 40-fold and (c) 80-fold synthesis upscale. Except the appearance of macroscopic agglomerates in a few cases, the nanobeads exhibit comparable diameters and reasonably narrow size distributions.*

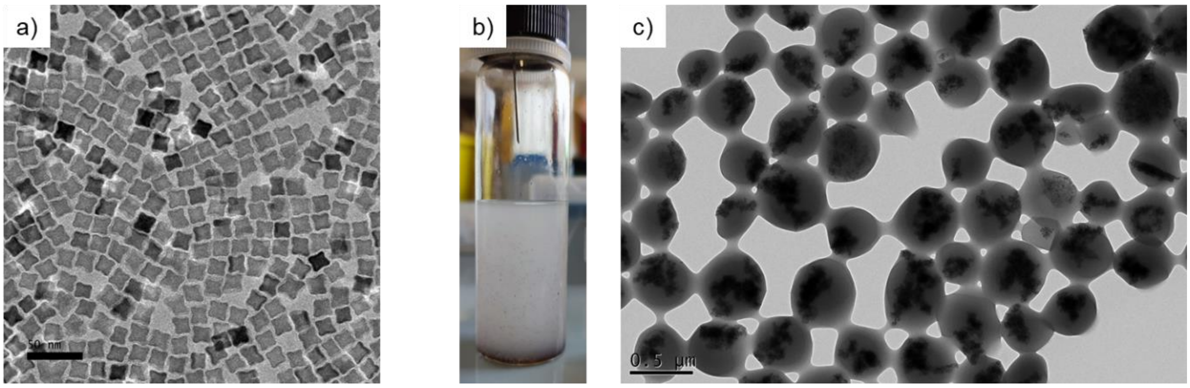

Figure S3. a) *TEM image of IONS, 13±1 nm in size, as deposited from a chloroform solution (scale bar 50 nm).* ***b)*** *Photograph of the IONS-MNBs solution obtained when applying the 100- fold protocol developed for Mn-IONP-MNBs. As shown in b), most of the magnetic material is precipitated out of the solution.* ***c)*** *Additional TEM images at low magnification of IONS-MNBs obtained by the ad hoc modified protocol.*

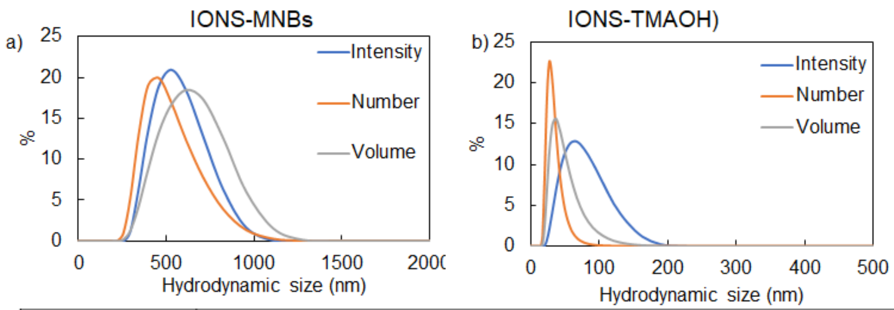

c)	Hydrodynamic size (nm)			
Sample	Intensity (%)	Number (%)	Volume (%)	PDI
IONS-MNBs	549±142	494±146	617±177	0.126
IONF-TMAOH	70±30	32±10	45±19	0.162

Figure S4: *Hydrodynamic size spectra (weighted by intensity, number and volume) of water soluble a) IONS-MNBs and b) IONS-TMAOH measured by DLS. c) The table summarizing all the hydrodynamic sizes and the corresponding full width half maximum of the peaks and the polydispersity index (PDI).*

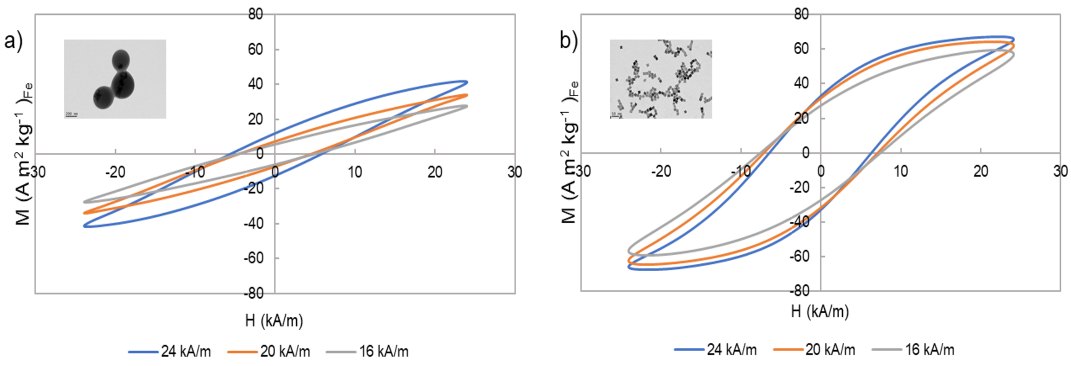

Figure S5. *AC hysteresis loops of a) IONS-MNBs and b) IONS measured at a frequency of 110 kHz and field range of 16-24 kA/m at a fixed iron concentration of 1 g/L in water.*

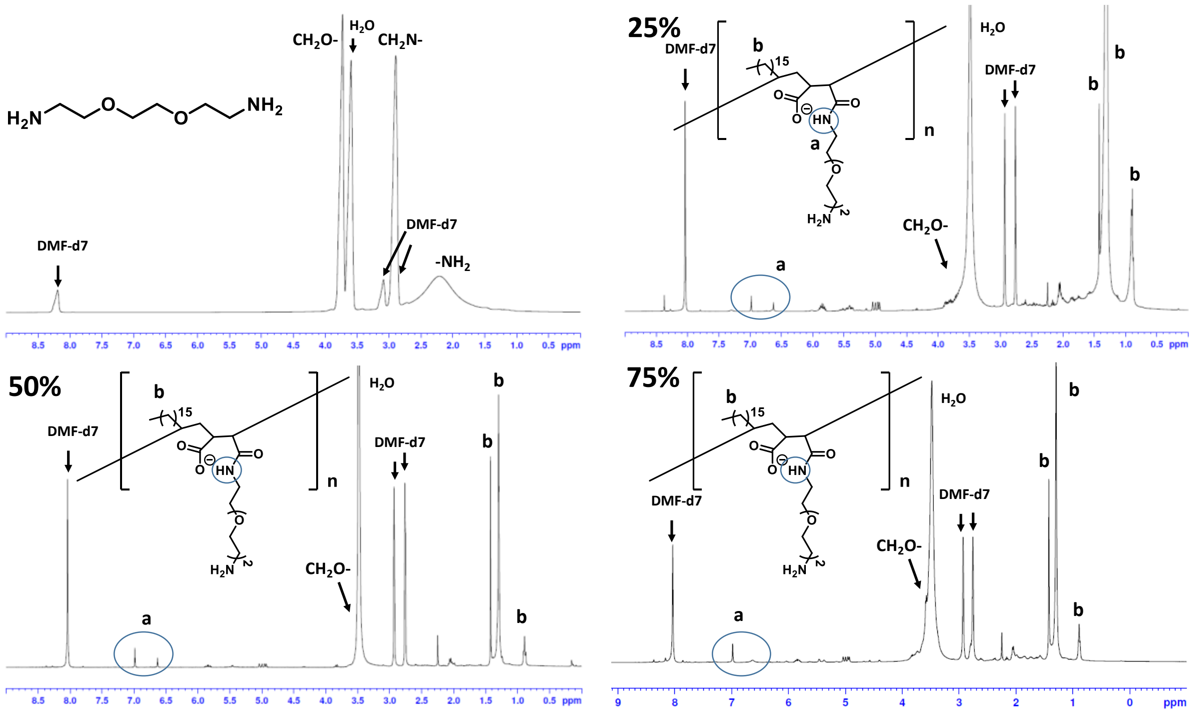

Figure S6. *^{1}H 400 MHz NMR spectra in DMF-d7 of (top left) the side chain molecule 2,2(ethylendedioxy)-bis-(ethylamine) attached to the PMA-OD for crosslinking the nanobeads, and of PMA-OD coupled with 25%, 50%, and 75% of the side chain confirming amide bond formation.*[SI 1]

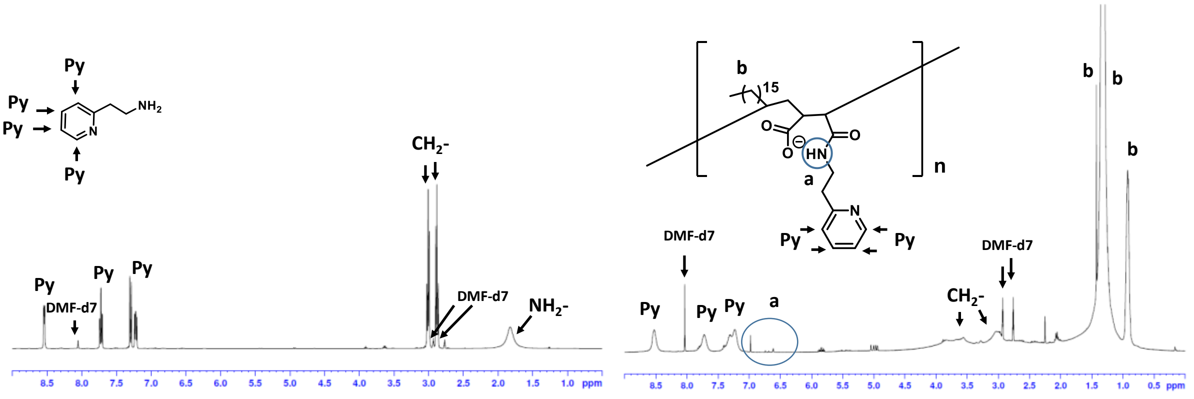

Figure S7. *^{1}H 400 MHz NMR spectra in DMF-d7 of (left) the side chain molecule derived pyridine attached to the PMA-OD for crosslinking the nanobeads, and of (right) PMA-OD coupled with 75% of the side chain confirming amide bond formation.*

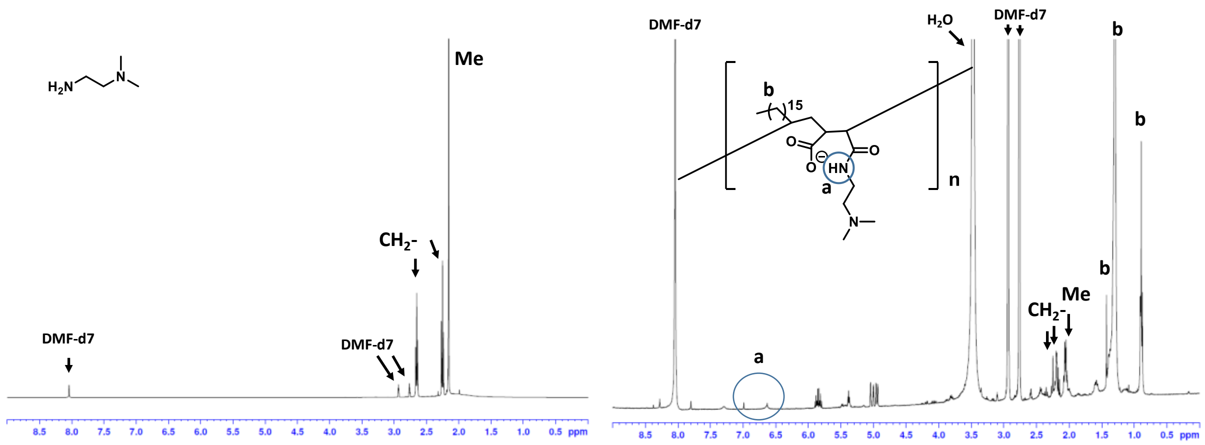

Figure S8. [1]*H 400 MHz NMR spectra in DMF-d7 of (left) the side chain molecule derived tertiary amine attached to the PMA-OD for crosslinking the nanobeads, and of (right) PMA-OD coupled with 75% of the side chain confirming amide bond formation.*

Beads Rxn. with	NPs Volume	PMA-OD (50 mM in THF)	THF Volume	Sonication Time/ Temperature	MillQ-water (Volume and flow rate) While shaking at 1000 rpm on a orbital shaker)	Sonication Time/ Temperature	Orbital Shaking Speed & Time	ACN solvent (Volume and flow rate) While shaking at 1000 rpm on a orbital shaker)
**Mn-IONPs***	220 μL	1.2 mL	3.66 mL	----	500 μL (8 mL/min)	-----	1000 rpm/ 45 min	16 mL (5 mL/min)
**IONS#**	313 μL	1.2 mL	3.66 mL	10 min/60°C	1000 μL (8 mL/min)	5 min/ 60°C	---	16 mL (5 mL/min)

- **Mn-IONPs* = 31.8 mg$_{Fe+Mn}$/mL.**
- **IONS# = 8.4 mg$_{Fe}$/mL.**

Table S1. *Summary of the reaction conditions used for the 110-fold scale up protocol of the magnetic nanobeads produced by Mn-IONPs or iron oxide nano stars (IONS).*

[SI 1] N.C. Bigall, A. Curcio, M. Pernia Leal, A. Falqui, D. Palumberi, R. Di Corato, R. Cingolani, T. Pellegrino, **Advanced Materials** 2011, *23*, 5645-5650.
